# Postoperative complications: an observational study of trends in the United States from 2012 to 2018

**DOI:** 10.1186/s12893-021-01392-z

**Published:** 2021-11-06

**Authors:** Emilie Even Dencker, Alexander Bonde, Anders Troelsen, Kartik Mangudi Varadarajan, Martin Sillesen

**Affiliations:** 1 Department of Surgical Gastroenterology and Transplantation C-TX, Copenhagen University Hospital, Blegdamsvej 9, Copenhagen Ø, 2100 Rigshospitalet, Denmark; 2 Center for Surgical Translational and Artificial Intelligence Research (CSTAR), Copenhagen University Hospital, Rigshospitalet, Denmark; 3 Department of Orthopedics, Copenhagen University Hospital, Hvidovre, Denmark; 4 Harris Orthopedics Laboratory, Massachusetts General Hospital, Boston, USA; 5 Institute of Clinical Medicine, University of Copenhagen, Hvidovre, Denmark

**Keywords:** Surgery, Complications, Trends

## Abstract

**Background:**

Postoperative complications continue to constitute a major issue for both the healthcare system and the individual patient and are associated with inferior outcomes and higher healthcare costs. The objective of this study was to evaluate the trends of postoperative complication rates over a 7-year period.

**Methods:**

The NSQIP datasets from 2012 to 2018 were used to assess 30-day complication incidence rates including mortality rate following surgical procedures within ten surgical subspecialties. Multivariable logistic regression was used to associate complication rates with dataset year, while adjusting for relevant confounders.

**Results:**

A total of 5,880,829 patients undergoing major surgery were included. Particularly the incidence rates of four complications were found to be decreasing: superficial SSI (1.9 to 1.3%), deep SSI (0.6 to 0.4%), urinary tract infection (1.6 to 1.2%) and patient unplanned return to the operating room (3.1 to 2.7%). Incidence rate for organ/space SSI exhibited an increase (1.1 to 1.5%). When adjusted, regression analyses indicated decreased odds ratios (OR) through the study period years for particularly deep SSI OR 0.92 [0.92–0.93], superficial SSI OR 0.94 [0.94–0.94] and acute renal failure OR 0.96 [0.95–0.96] as the predictor variable (study year) increased (p < 0.01). However, OR’s for organ/space SSI 1.05 [1.05–1.06], myocardial infarction 1.01 [1.01–1.02] and sepsis 1.01 [1.01–1.02] increased slightly over time (all p < 0.01).

**Conclusions:**

Incidence rates for the complications exhibited a stable trend over the study period, with minor in or decreases observed.

**Supplementary Information:**

The online version contains supplementary material available at 10.1186/s12893-021-01392-z.

## Background

The total volume of major surgeries performed annually worldwide was estimated to be 312.9 million in 2012—an increase of 38.2% from 2004 [1]. Studies estimate that 7–15% of patients undergoing major surgery will experience a postoperative complication (PC) [2, 3]. Furthermore, the overall postoperative mortality rate is reported to vary from 0.79 to 5.7% [4]. According to the Clavien-Dindo classification, a PC is defined as any deviation from the normal postoperative course, meaning that the severity ranges from non-life-threatening complications with no lasting disability to fatal outcomes [5, 6]. In the individual level, PCs can have major impact on the individual patient, potentially resulting in a decline of both quality of life as well as functional performance [4]. From a societal standpoint, PCs account for a large financial burden in the form of additional health care expenses when a patient requires Intensive Care Unit (ICU) treatment, reoperation or readmission, thereby increasing healthcare costs [5, 7, 8] in an estimated order of magnitude between $11,626 and $19,626 in additional healthcare expenses per patient [7, 9].

A number of studies have highlighted the scope of the problem within specific surgical subspecialties. PC trends following total knee arthroplasty (TKA) has been reported to decrease during a 10-year study period (2006–2016) [10]. However, studies assessing this issue in general across multiple subspecialties and thereby estimating the overall extent of postoperative complications and its trend over time are lacking. The primary objective in this study was to evaluate the trends for postoperative complication rates over a 7-year period (2012–2018). Our secondary objective was to assess whether complications incidences exhibited significant changes over the study period.

We hypothesized that the rates of incidence for fourteen specified postoperative complications would exhibit minor changes over this 7-year period.

## Methods

This study utilized the American College of Surgeons (ACS) National Surgical Quality Improvement Program (NSQIP). NSQIP is a national database containing data on multiple surgical outcomes from more than 680 participating hospital sites in the United States and data in the NSQIP database are captured by a site’s trained and certified Surgical Clinical Reviewer (SCR) [11]. The study was approved by the ACS and the Massachusetts General Hospital Institutional Review Board. Findings are reported in accordance with STROBE guidelines.

### Patient selection

**Fig. 1:**
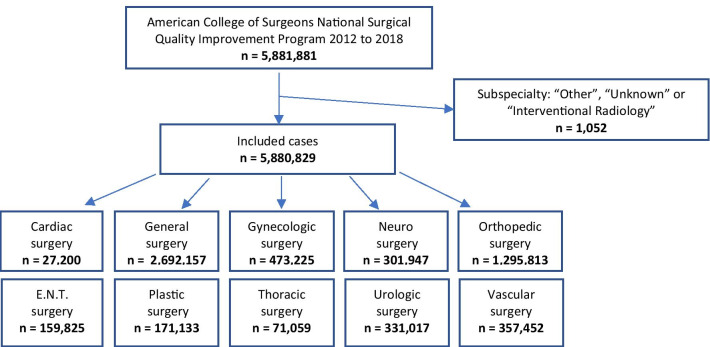
Flowchart of patient cases from American College of Surgeons (ACS) National Surgical Quality Improvement Program (NSQIP) included in the study. All cases with available information on sex, age and surgical subspecialty were included

We included patients admitted from the period January 1st, 2012 to December 31st, 2018. All patients were > 18 years. Patients with subspecialty listed as “Other”, “Unknown” or “Interventional radiology” were excluded from the study (Fig. 1). In the NSQIP data, maximum age was coded as 90+. This was recoded as 90 years of age in our calculations to enable calculation of median age. Elective as well as emergency cases were included. Preoperative variables were collected including demographics, comorbidities and surgical subspecialty. We identified patients with any of the following clinical outcomes occurring within 30 days of the index procedure: superficial surgical site infection (SSI), deep incisional SSI, organ/space SSI, pneumonia, pulmonary embolism, acute renal failure, urinary tract infection, Cerebrovascular Accident (CVA)/stroke with neurological deficit, cardiac arrest requiring cardiopulmonary resuscitation (CPR), Myocardial Infarction (MI), deep venous thrombosis (DVT), sepsis/septic shock, and unplanned return to the operating room (OR). All cases of 30-day postoperative all-cause mortality were collected as well. Outcomes were defined by NSQIP and registered by the NSQIP SCRs [12].

### Statistical analysis

Descriptive statistics were performed to describe demographics, postoperative morbidity and mortality from 2012 to 2018. We calculated postoperative complication incidence rates over the study period for each outcome within the various subspecialties. Data are presented as medians with interquartile range, or percentages for dichotomous variables where appropriate. A logistic regression model was used to associate complication incidences with study year, the latter modelled as a continuous variable. This approach was chosen in order to allow for an assessment of whether the slope of the incidence curve in or decreased over the study years when adjusting for relevant confounders. Data are presented as odds ratios (OR) with [95% confidence intervals (CI)] for each postoperative complication. Analyses results are presented as univariable as well as multivariable regression, adjusted for the variables: surgical subspecialty, age, gender, body mass index (BMI), American Society of Anesthesiology (ASA) score, diabetes (including both insulin and non-insulin requiring), chronic obstructive pulmonary disease (COPD), blood transfusion, smoking, preoperative renal failure and hypertension requiring medication.

The choice of these covariates was based on the desire to include covariates with a reasonably documented association with PCs and thus model overall patient frailty. We opted to use this approach as opposed to a data driven approach (e.g., stepwise regression), as studies have indicated a frequent over-inclusion of covariates in disagreement with expert consensus using this approach [13].

BMI was calculated using the formula BMI = weight (lb)/[height (in)]2 × 703.

All statistical analysis was performed in R version 3.5.2 [14]. Regression modelling was made using the standard R glm approach. A p-value < 0.05 was considered statistically significant.

### Missing data

Missing data was assumed to be missing at random and was handled by listwise exclusion in the multivariable modelling. An overview of missing data is presented in Additional file 1: Table S1 and comprised of < 0.001% of the total cohort. As such, missing data was not considered to have relevant impact on the dataset, and imputation was thus not done.

## Results

A total of 5,880,820 patients who underwent major surgery from 2012 to 2018 were included in the study (Table 1 and Fig. 1). The most common of the included comorbidities (Table 1) was hypertension (44.9%), followed by diabetes (15.5%) and Chronic Obstructive Pulmonary Disease (COPD, 4.4%). Furthermore, 5.4% of patients experienced sepsis preoperatively. General surgery accounted for the majority of the surgical procedures with a total of 2,692,157 (45.8%) performed procedures followed by orthopedic surgery (22.0%) (Table 1 and Fig. 1). Median ASA scores were 2 for all included study years.

**Table 1:** Demographics of the 5,880,829 surgical cases from ACS NSQIP from 2012–2018. Values are median [Interquartile Range] or number/proportion

Procedure year	2012	2013	2014	2015	2016	2017	2018	All
Individuals	543,815	651,770	750,792	885,350	1,000,195	1,028,574	1,020,333	5,880,829
Age; years	58 [45–69]	58 [45–69]	58 [44–69]	58 [45–69]	58 [45–69]	58 [45–69]	59 [45–70]	58 [45–69]
Sex; male	232,164 (42.7%)	276,715 (42.5%)	324,125 (43.2%)	384,005 (43.4%)	434,783 (43.5%)	443,156 (43.1%)	437,897 (42.9%)	2,532,845 (43.1%)
BMI; kg/m^2^	28.3 [24.4–33.5]	28.4 [24.4–33.7]	28.7 [24.5–33.8]	28.7 [24.7–33.9]	28.9 [24.8–34.0]	28.9 [24.9–34.1]	28.9 [24.9–34.0]	28.6 [24.8–33.9]
Surgery duration	86 min [50–144]	86 min [50–143]	86 min [50–142]	84 min [50–139]	84 min [50–138]	85 min [51–139]	85 min (51–141)	85 min [50–140]
Ethnicity:								
American Indian/ Alaska Native	2,639 (0.5%)	4,205 (0.6%)	5,390 (0.7%)	5,563 (0.6%)	5,408 (0.5%)	5,109 (0.5%)	5,145 (0.5%)	33,459 (0.6%)
Asian	16,423 (3.0%)	18,845 (2.9%)	21,080 (2.8%)	24,002 (2.7%)	26,504 (2.6%)	29,518 (2.9%)	30,423 (3.0%)	166,795 (2.8%)
Black or African American	50,557 (9.3%)	62,767 (9.6%)	75,877 (10.1%)	88,936 (10.0%)	100,507 (10.0%)	105,298 (10.2%)	102,018 (10.0%)	585,960 (10.0%)
Hispanic	5,688 (1.0%)	8,436 (1.3%)	11,258 (1.5%)	13,635 (1.5%)	17,953 (1.8%)	22,131 (2.2%)	24,678 (2.4%)	103,779 (1.8%)
Native Hawaiian/ Pacific Islander	2,336 (0.4%)	2.722 (0.4%)	3,103 (0.4%)	3,469 (0.4%)	3,768 (0.4%)	3,762 (0.4%)	3,616 (0.4%)	22,776 (0.4%)
White	403,719 (74.2%)	483,949 (74.3%)	551,803 (73.5%)	640,518 (72.3%)	705,368 (70.5%)	722,782 (70.3%)	700,901 (68.7%)	4,209,040 (71.6%)
Unknown/ Not reported	62,453 (11.5%)	70,844 (10.9%)	82,281 (11.0%)	109,227 (12.3%)	140,687 (14.1%)	139,974 (13.6%)	153,552 (15.0%)	759,018 12.9%
Comorbidities								
Hypertension requiring medication	249,610 (45.9%)	298,722 (45.8%)	337,796 (45.0%)	398,197 (45.0%)	447,427 (44.7%)	458,267 (44.5%)	448,949 (44.0%)	2,638,968 (44.9%)
Diabetes	82,348 (15.1%)	99,985 (15.3%)	114,839 (15.3%)	136,865 (15.5%)	156,468 (15.6%)	160,816 (15.6%)	157,897 (15.5%)	909,218 (15.5%)
COPD	25,054 (4.6%)	30,229 (4.6%)	34,304 (4.6%)	40,980 (4.6%)	43,711 (4.4%)	43,623 (4.2%)	42,115 (4.1%)	260,016 (4.4%)
Ascites	2,495 (0.5%)	2,616 (0.4%)	2,832 (0.4%)	3,182 (0.4%)	3,016 (0.3%)	2,991 (0.3%)	2,896 (0.3%)	20,028 (0.3%)
Congestive heart failure	4,119 (0.8%)	5,421 (0.8%)	6,626 (0.9%)	8,221 (0.9%)	8,800 (0.9%)	8,538 (0.8%)	8,548 (0.8%)	50,273 (0.9%)
Dialysis	7,631 (1.4%)	9,062 (1.4%)	10,101 (1.3%)	11,985 (1.4%)	13,070 (1.3%)	13,206 (1.3%)	13,049 (1.3%)	78,104 (1.3%)
Disseminated cancer	11,287 (2.1%)	14,846 (2.3%)	17,846 (2.3%)	20,472 (2.3%)	22,355 (2.2%)	23,566 (2.3%)	23,252 (2.3%)	133,624 (2.3%)
Sepsis	29,103 (5.4%)	33,796 (5.2%)	39,512 (5.3%)	47,468 (5.4%)	54,688 (5.5%)	57,643 (5.6%)	57,067 (5.6%)	319,277 (5.4%)
Surgical subspecialty:								
Cardiac	4,028 (0.7%)	3,093 (0.5%)	3,693 (0.5%)	4,033 (0.5%)	4,281 (0.4%)	4,012 (0.4%)	4,060 (0.4%)	27,200 (0.5%)
General surgery	277,926 (51.1%)	322,058 (49.4%)	360,397 (48.0%)	409,231 (46.2%)	445,639 (44.5%)	444,203 (43.2%)	432,703 (42.4%)	2,692,157 (45.8%)
Gynecology	36,941 (6.8%)	46,645 (7.2%)	55,339 (7.4%)	65,653 (7.4%)	77,744 (7.8%)	92,077 (9.0%)	98,826 (9.7%)	473,225 (8.0%)
Neurosurgery	23,585 (4.3%)	32,640 (5.0%)	37,442 (5.0%)	46,665 (5.3%)	53,127 (5.3%)	54,856 (5.3%)	53,632 (5.3%)	301,947 (5.1%)
Orthopedics	92,592 (17.0%)	122,036 (18.7%)	153,320 (20.4%)	197,868 (22.3%)	235,029 (23.5%)	243,991 (23.7%)	250,977 (24.6%)	1,295,813 (22.0%)
Otolaryngology (ENT)	14,621 (2.7%)	17,001 (2.6%)	20,859 (2.8%)	24,658 (2.8%)	28,147 (2.8%)	27,891 (2.7%)	26,648 (2.6%)	159,825 (2.7%)
Plastics	14,777 (2.7%)	18,440 (2.8%)	21,108 (2.8%)	24,596 (2.8%)	29,382 (2.9%)	31,124 (3.0%)	31,706 (3.1%)	171,133 (2.9%)
Thoracic	7397 (1.4%)	8375 (1.3%)	9420 (1.3%)	9788 (1.1%)	11,323 (1.1%)	12,298 (1.2%)	12,458 (1.2%)	71,059 (1.2%)
Urology	29,044 (5.3%)	34,015 (5.2%)	39,632 (5.3%)	49,184 (5.6%)	57,963 (5.8%)	61,140 (5.9%)	60,039 (5.9%)	331,017 (5.6%)
Vascular	42,904 (7.9%)	47,467 (7.3%)	49,582 (6.6%)	53,674 (6.1%)	57,560 (5.8%)	56,981 (5.5%)	49,284 (4.8%)	357,452 (6.1%)

*COPD* chronic obstructive pulmonary disease, *ENT* ear-nose-throat, *BMI* body mass index

**Fig. 2:**
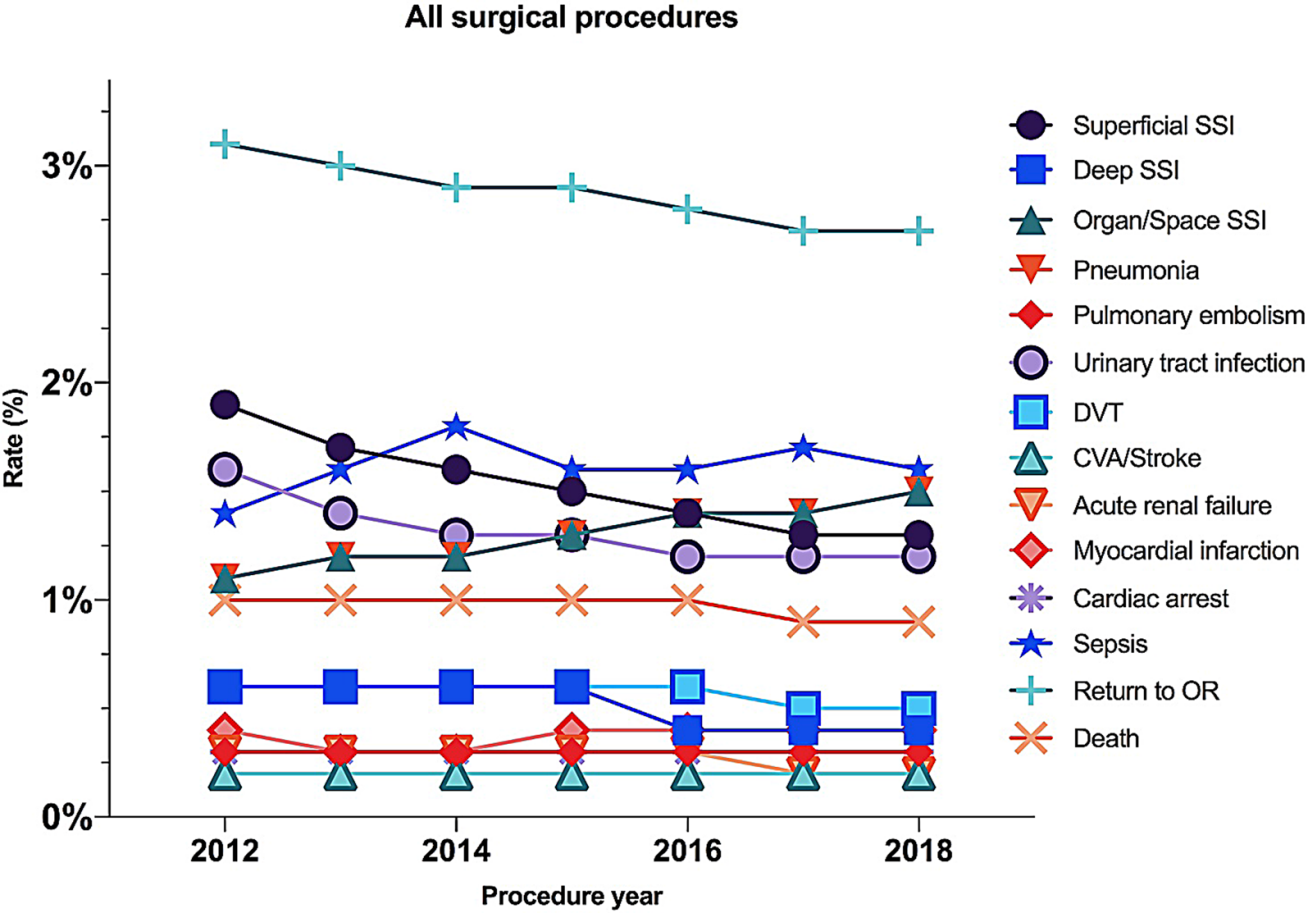
Incidence rates (%) over the study period for the 14 studied postoperative complications for all surgical procedures combined. *SSI* surgical site infection; *DVT* deep venous thrombosis, *CVA* cerebrovascular accident, *OR* operating room

Postoperative complication incidences and rates for all surgical procedures combined are shown in Table 2. From 2012 to 2018 the incidence rates for the following complications appear decreasing: superficial SSI (1.9 to 1.3%), deep SSI (0.6 to 0.4%), urinary tract infection (1.6 to 1.2%) and patient unplanned return to the operating room (3.1 to 2.7%). The incidence rate for postoperative organ/space SSI for all surgical procedures combined exhibit an increase (1.1 to 1.5%). We observed no relevant changes in the nine remaining studied PC incidence rates within the study period (Table 2 and Fig. 2).

**Table 2:** Incidence rates for the 14 studied postoperative complications for all surgical procedures combined

Procedure year	2012	2013	2014	2015	2016	2017	2018	All
All surgical procedures**:**								
Unplanned return to OR:	**3.1%** (16,983)	**3.0%** (19,730)	**2.9%** (21,822)	**2.9%** (25,875)	**2.8%** (27,548)	**2.7%** (27,916)	**2.7%** (27,260)	**2.8%**(167,134)
Sepsis:	**1.4%** (7,750)	**1.6%** (10,169)	**1.8%** (13,305)	**1.6%** (14,593)	**1.6%** (16,366)	**1.7%** (17,250)	**1.6%** (16,296)	**1.6%** (95,729)
Superficial SSI:	**1.9%** (10,062)	**1.7%** (11,065)	**1.6%** (12,195)	**1.5%** (13,072)	**1.4%** (14,077)	**1.3%** (13,713)	**1.3%** (13,269)	**1.5%** (87,453)
Organ/Space SSI:	**1.1%** (5,775)	**1.2%** (7542)	**1.2%** (9305)	**1.3%** (11,146)	**1.4%** (13,578)	**1.4%** (14,664)	**1.5%** (14,986)	**1.3%** (76,996)
Urinary tract infection:	**1.6%** (8,466)	**1.4%** (9162)	**1.3%** (9,96)	**1.3%** (11,606)	**1.2%** (12,166)	**1.2%** (12,227)	**1.2%** (12,345)	**1.3%** (75,968)
Pneumonia:	**1.1%** (6,195)	**1.2%** (8052)	**1.3%** (9732)	**1.3%** (11,310)	**1.2%** (11,831)	**1.1%** (11,449)	**1.0%** (10,498)	**1.2%** (69,067)
Death:	**1.0%** (5,640)	**1.0%** (6756)	**1.0%** (7749)	**1.0%** (8983)	**1.0%** (9732)	**0.9%** (9664)	**0.9%** (9546)	**1.0%** (58,070)
DVT/Thrombo-phlebitis:	**0.6%** (3,315)	**0.6%** (3887)	**0.6%** (4336)	**0.6%** (5090)	**0.6%** (5511)	**0.5%** (5626)	**0.5%** (5242)	**0.6%** (33,007)
Deep SSI:	**0.6%** (3,149)	**0.6%** (4121)	**0.6%** (4846)	**0.6%** (5085)	**0.4%** (4226)	**0.4%** (3960)	**0.4%** (3779)	**0.5%** (29,166)
Myocardial infarction:	**0.4%** (1,997)	**0.3%** (2102)	**0.3%** (2617)	**0.4%** (3338)	**0.4%** (3792)	**0.4%** (3853)	**0.4%** (3607)	**0.4%** (21,306)
Cardiac arrest:	**0.3%** (1,616)	**0.3%** (2003)	**0.3%** (2351)	**0.3%** (2824)	**0.3%** (2957)	**0.3%** (3056)	**0.3%** (2859)	**0.3%** (17,666)
Acute renal fail:	**0.3%** (1,730)	**0.3%** (1946)	**0.3%** (2178)	**0.3%** (2410)	**0.3%** (2577)	**0.2%** (2469)	**0.2%** (2448)	**0.3%** (15,758)
Pulmonary embolism:	**0.3%** (1,800)	**0.3%** (2132)	**0.3%** (2514)	**0.3%** (3066)	**0.3%** (3229)	**0.3%** (3367)	**0.3%** (3179)	**0.3%** (19,287)
CVA/Stroke:	**0.2%** (1,083)	**0.2%** (1318)	**0.2%** (1414)	**0.2%** (1742)	**0.2%** (1981)	**0.2%** (1970)	**0.2%** (1843)	**0.2%** (11,351)
Total complication rate:	**13.9%** (75,561)	**13.8%** (89,985)	**13.9%** (104,360)	**13.6%** (120,140)	**13.0%** (129,571)	**12.8%** (131,184)	**12.5%** (127,157)	**13.2%** (777,958)

Bold font indicates incidence rates for the 14 studied postoperative complications for all surgical procedures combined

Values are listed by procedure year as incidence (%) and number of cases

*OR* operating room, *SSI* surgical site infection, *DVT* deep venous thrombosis, *CVA* cerebrovascular accident

**Fig. 3:**
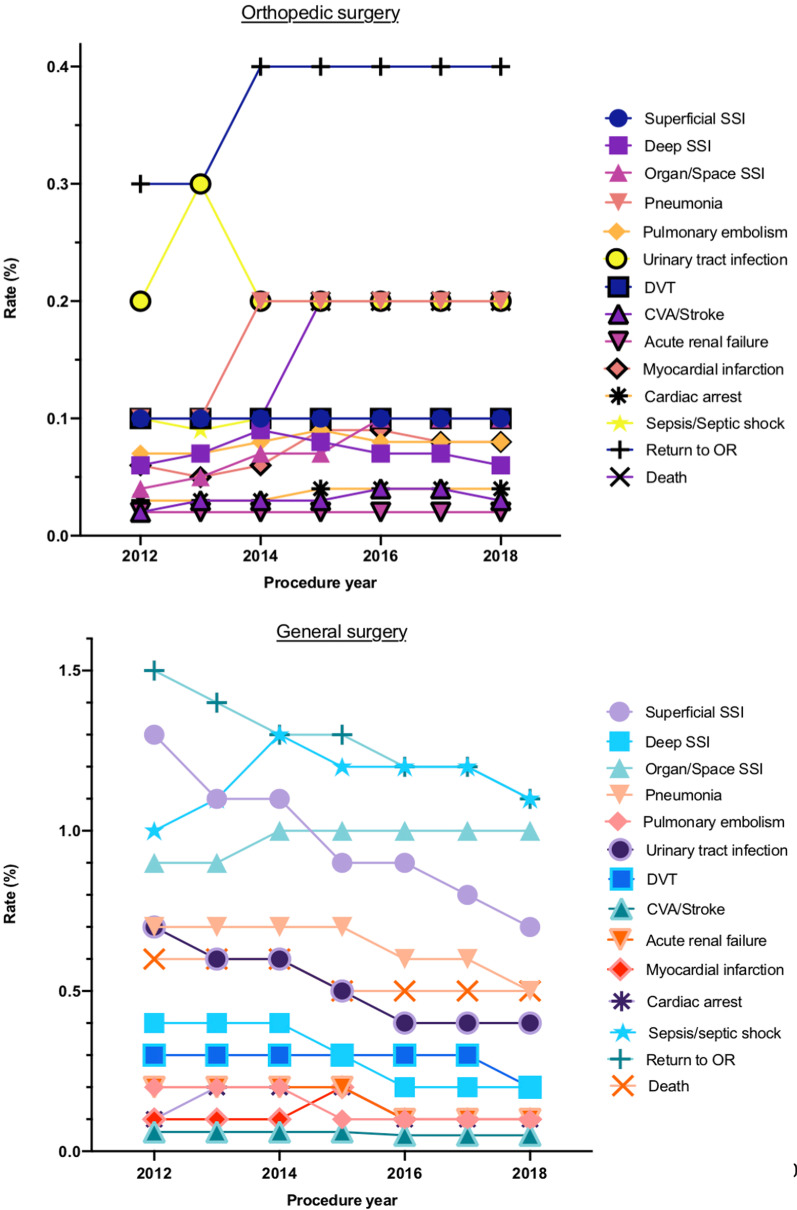
Postoperative complication incidence rates from 2012 to 2018 for orthopedic (top) and general (bottom) surgery. SSI: Surgical Site Infection; DVT: Deep Venous Thrombosis; CVA: Cerebrovascular Accident: OR: Operating Room

Overall, similar trends were generally found when stratified into surgical subspecialties and are presented in Additional file 1: Table S2). Figure 3 illustrates the trends for all studied complications within the two largest subspecialties: general and orthopedic surgery.

Results of both the univariable and multivariable regression model are show in Table 3. When corrected for relevant confounders, regression analyses indicated decreased odds ratios (OR) for the majority of the studied complications—most apparent for deep SSI OR 0.92 [0.92–0.93], superficial SSI OR 0.94 [0.94–0.94] and acute renal failure OR 0.96 [0.95–0.96], as the predictor variable (study year) increased (p < 0.01). In contrast, OR for organ/space SSI, MI and sepsis increased over time (Table 3).

**Table 3:** Results of the regression models; a univariable model and a corrected model (corrected for sex, age, Body Mass Index and American Society of Anesthesia (ASA)-classification score, diabetes, chronic obstructive pulmonary disease (COPD), blood transfusion, smoking, preoperative renal failure hypertension requiring medication and surgical subspecialty

Complications	Corrected model			Univariate model		
	Odds Ratio	95% CI	P-value	Odds Ratio	95% CI	P-value
Organ/space surgical site infection	1.05	[1.05–1.06]	< 0.01	1.05	[1.05–1.06]	< 0.01
Myocardial infarction	1.01	[1.01–1.02]	< 0.01	1.01	[1.00–1.02]	0.01
Sepsis/Septic Shock	1.01	[1.01–1.01]	< 0.01	1.01	[1.01–1.02]	< 0.01
Pulmonary embolism	0.99	[0.98–0.99]	< 0.01	0.99	[0.98–1.00]	0.01
Cardiac arrest requiring CPR	0.98	[0.98–0.99]	< 0.01	0.99	[0.98–0.99]	< 0.01
CVA/Stroke w. neurological deficit	0.99	[0.98–1.00]	< 0.01	0.99	[0.98–1.00]	< 0.01
Pneumonia	0.98	[0.97–0.98]	< 0.01	0.97	[0.97–0.98]	< 0.01
Death	0.98	[0.97–0.98]	< 0.01	0.98	[0.97–0.98]	< 0,01
Unplanned return to OR	0.98	[0.98–0.99]	< 0.01	0.97	[0.97–0.98]	< 0.01
DVT/thrombophlebitis	0.97	[0.97–0.98]	< 0.01	0.97	[0.97–0.98]	< 0.01
Urinary tract infection	0.96	[0.95–0.96]	< 0.01	0.96	[0.96–0.96]	< 0.01
Acute renal fail	0.96	[0.95–0.96]	< 0.01	0.95	[0.94–0.96]	< 0.01
Superficial surgical site infection	0.94	[0.94–0.94]	< 0.01	0.94	[0.94–0.94]	< 0.01
Deep surgical site infection	0.92	[0.92–0.93]	< 0.01	0.90	[0.90–0.91]	< 0.01

Odds ratios (OR) and 95% confidence intervals and p-values are listed. Odds Ratio’s represent results from the logistical regression model associating the occurrence of a given complication with study year whilst correcting for confounders

*DVT* deep venous thrombosis, *CVA* cerebrovascular accident, *OR* operating room

Odds ratios for the remaining studied complications were all statistically significant with p-values < 0.05 but exhibited minor decreases or increases in OR (Table 3).

## Discussion

In this study we investigated the development of complication incidences following surgical procedures in the US as registered in the NSQIP database from 2012 to 2018. We found that several complication incidences exhibited a temporal decline during the study period, in which superficial SSI, deep SSI, urinary tract infection and unplanned return to operating room appear to have the most declining rates. Meanwhile the incidence rate for organ/space SSI increased (Table 2). These findings were largely consistent throughout various subspecialties (Additional file 1: Table S2).

In 2014 more than 14 million operating room procedures were performed during inpatient hospital stays in the US, as reported by the Agency for Healthcare Research and Quality (AHRQ) [15]. In addition, AHRQ reported 17.3 million outpatient surgeries in 2012[16]. The volume of surgical procedures performed annually in the U.S. could therefore exceed 30 million in total [17]. The NSQIP database includes major surgical procedures in both the inpatient and outpatient setting [18]. If the results of this study can be extrapolated to national practice in the US, a total complication incidence rate of 12.5% in 2018 (Table 2) applied to the estimated number of nationwide surgical procedures would mean that 3,750,000 postoperative complications occurred in the US in 2018. This estimate is in all probability lower than the actual number of postoperative complications as this study only includes fourteen different complications. According to Dimick et al., the mean hospital costs were estimated to be $11,626 higher for patients with major postoperative complications, while Healy et al. estimated the additional healthcare costs caused by complications to be $19,626 [7, 9]. This would correspond to the expense of $43–73 billion in additional healthcare costs caused by postoperative complications.

Overall, the literature on postoperative complication trends across surgical subspecialities is limited. The majority of studies focus on either surgical subspecialty, type of complication or both and reported results vary. A recent NSQIP-based study shows trends of decreasing incidence rates for various complications following total knee arthroplasty with a decrease in total complication rates in the most recent cohort (2014–2016) compared to the 2006–2009 cohort (OR 0.70) [10]. Another NSQIP study examining complications following colorectal surgery also found declining rates for most complications including SSI, urinary tract infection, sepsis, DVT, re-operation and mortality [19].

On the contrary, a study of trends in surgical outcomes for rectal cancer found no significant improvement in morbidity or mortality for the two studied cohorts over a 12-year period [20]. An earlier study (2002) of 4700 inpatient procedures reported considerably higher complication rates (overall 32.1%, varying from 26.9 to 42.4%) within four subspecialties [21].

The large span within results in the literature could have several explanations—most obvious the study design. The above-mentioned studies included between 4700 and 310,000 cases, some within a highly specialized surgical field. In contrast, this study includes over 6 million patients from an array of subspecialities, which could in part account for the difference in findings. We did, however, find a statistically significant decrease of several complications, most noticeable for deep SSI, superficial SSI, acute renal failure and urinary tract infection and minor decreases for DVT and pneumonia (Table 3), which agrees fairly with some previous findings.

Part of these declining rates could be associated with increased adherence to pre-emptive strategies for prophylactic antibiotics, thrombosis prophylaxis and sepsis control management etc. [22, 23], although studies have suggested that prophylactic guidelines are often not followed when real-world data is analyzed [24].

It is, however, interesting to note that the incidence rates for organ/space SSI, MI and sepsis do not exhibit a decrease in OR over time when corrected for comorbidities.

A reason for this slight increase in OR for organ/space SSI over time could in part be due to the increasing proportion of minimal invasive procedures compared to open procedures in abdominal surgery. This would most likely reduce the number of superficial SSIs and potentially also deep SSIs, but not affect the number of organ/space SSIs.

The observed results for sepsis mirror reports indicating that incidence rates are stable or somewhat increasing (depending on criteria/claims) from 2009 to 2014 [25]. Results regarding MI incidences seem to be conflicting with reports indicating an overall reduced incidence in the general population [26, 27]. Although this remains speculative, the presented results could indicate an increase in the diagnostic precision of MI rather than an actual increase in incidence.

Finally, it is important to note that although we observed variations in incidences of various complications, we cannot conclude on whether this is due to changes in treatment practices or changes in the underlying patient comorbidity profiles over time.

Although incidence rates of all complications exhibit significant developments over the study period (Table 3), these findings should be interpreted with caution. Due to the large patient cohort, even smaller variations will be statistically significant when performing regression analyses, without constituting an actual clinically important decline or increase. Indeed, variations in complication incidences over the study period were minor, collectively indicating that despite recent efforts in reducing complication rates, a somewhat stable subset of patients remain refractory to various prophylactic treatment efforts. This issue is again highlighted by the presented odds ratios, indicating that the overall magnitude of the effect was limited, even though findings were significant.

These patients could potentially benefit from novel precision medicine-based approaches aimed at identifying high-risk patients prior to surgery, although these approaches would require extensive validation efforts followed by large scale clinical trial efforts. To our knowledge, such trials targeting common complications in diverse surgical cohorts, have yet to be fielded.

This study provides high quality, multi-centered data and is based on a large patient cohort, yet it has its limitations. First, NSQIP complications are only registered during the 30-day follow-up period. This might result in an underestimation of the actual complication incidence rates. Secondly, NSQIP participation hospitals are often large academic centers, where complication rates could be different than non-academic facilities. This would result in data not being representative for the entire country and the incidence rates would in all probability be higher than reported in this study.

Third, as the study design was retrospective, we were not able to examine the cause/effect relationships and consequently this study will provide associations, but not causality.

## Conclusion

In conclusion the incidence rates for the studied complications exhibit a somewhat stable trend comprising smaller changes during the study period. Future research should address how to identify patients at risk for PCs—possibly by applying personalized medicine for pre- and perioperative risk assessment.

## Supplementary Information

**Additional file 1: Table S1.** Missing data for variables “Sex”, “Age”,” Subspecialty” and the fourteen studied complications. Values are listed by procedure year as number of missing data and percentage for the year in question (%). **Table S2.** Incidence rates for the 14 studied postoperative complications stratified into surgical subspecialties.

## Data Availability

Data available to participating member hospitals of the ACS NSQIP upon reasonable request.

## Abbreviations

NSQIP: National Surgical Quality Improvement Program
PC: Postoperative complication
SSI: Surgical site infection
DVT: Deep venous thrombosis
OR: Odds ratio
AHRQ: Agency for Healthcare Research and Quality
ACS: American College of Surgeons
SCR: Surgical risk calculator
COPD: Chronic obstructive pulmonary disease
