# Supplementary material for: Postoperative complications: an observational study of trends in the United States from 2012 to 2018
Source: BMC Surg. 2021 Nov 6;21:393. doi: 10.1186/s12893-021-01392-z (PMC8571843; doi:10.1186/s12893-021-01392-z)
Supplement: Supplementary file 1 — Additional file 1: Table S1. Missing data for variables “Sex”, “Age”,” Subspecialty” and the fourteen studied complications. Values are listed by procedure year as number of missing data and percentage for the year in question (%). Table S2. Incidence rates for the 14 studied postoperative complications stratified into surgical subspecialties. [file 12893_2021_1392_MOESM1_ESM.docx]

**Additional tables**

**Additional file 1: Table S1** Missing data for variables “Sex”, “Age”,” Subspecialty” and the fourteen studied complications. Values are listed by procedure year as number of missing data and percentage for the year in question (%).

SSI: Surgical Site Infection; UTI: Urinary Tract Infection; DVT: Deep Venous Thrombosis; CVA: Cerebrovascular Accident; OR: Operating Room

| **Procedure year** | **2012** | **2013** | **2014** | **2015** | **2016** | **2017** | **2018** | **Total per variable** |
| --- | --- | --- | --- | --- | --- | --- | --- | --- |
| **Sex:** | 0  (0%) | 0  (0%) | 1  (0.0001%) | 0  (0%) | 0  (0%) | 0 (0%) | 3  (0.0003%) | 4  (0.00007 %) |
| **Age:** | 1  (0.0002%) | 6  (0.0009%) | 0 (0%) | 1  (0.0001%) | 0  (0%) | 0  (0%) | 0  (0%) | 8  (0.0001%) |
| **Subspecialty:** | 0  (0%) | 0  (0%) | 0  (0%) | 0  (0%) | 0  (0%) | 1  (0.00009%) | 0  (0%) | 1  (0.00002%) |
| **Superficial SSI:** | 0 (0%) | 0 (0%) | 0 (0%) | 0 (0%) | 0 (0%) | 0 (0%) | 0 (0%) | 0 (0%) |
| **Deep SSI:** | 0 (0%) | 0 (0%) | 0 (0%) | 0 (0%) | 0 (0%) | 0 (0%) | 0 (0%) | 0 (0%) |
| **Organ/Space SSI:** | 0 (0%) | 0 (0%) | 0 (0%) | 0 (0%) | 0 (0%) | 0 (0%) | 0 (0%) | 0 (0%) |
| **Pneumonia:** | 0 (0%) | 0 (0%) | 0 (0%) | 0 (0%) | 0 (0%) | 0 (0%) | 0 (0%) | 0 (0%) |
| **Pulmonary embolism:** | 0 (0%) | 0 (0%) | 0 (0%) | 0 (0%) | 0 (0%) | 0 (0%) | 0 (0%) | 0 (0%) |
| **UTI:** | 0 (0%) | 0 (0%) | 0 (0%) | 0 (0%) | 0 (0%) | 0 (0%) | 0 (0%) | 0 (0%) |
| **DVT:** | 0 (0%) | 0 (0%) | 0 (0%) | 0 (0%) | 0 (0%) | 0 (0%) | 0 (0%) | 0 (0%) |
| **CVA/Stroke:** | 0 (0%) | 0 (0%) | 0 (0%) | 0 (0%) | 0 (0%) | 0 (0%) | 0 (0%) | 0 (0%) |
| **Acute Renal Fail:** | 0 (0%) | 0 (0%) | 0 (0%) | 0 (0%) | 0 (0%) | 0 (0%) | 0 (0%) | 0 (0%) |
| **Myocardial infarction:** | 0 (0%) | 0 (0%) | 0 (0%) | 0 (0%) | 0 (0%) | 0 (0%) | 0 (0%) | 0 (0%) |
| **Cardiac Arrest:** | 0 (0%) | 0 (0%) | 0 (0%) | 0 (0%) | 0 (0%) | 0 (0%) | 0 (0%) | 0 (0%) |
| **Sepsis:** | 0 (0%) | 0 (0%) | 0 (0%) | 0 (0%) | 0 (0%) | 0 (0%) | 0 (0%) | 0 (0%) |
| **Unplanned return OR:** | 1  (0.0002%) | 3  (0.0005%) | 0  (0%) | 0  (0%) | 0  (0%) | 0  (0%) | 0  (0%) | 4  (0.00007 %) |
| **Mortality:** | 36  (0.007%) | 0  (0%) | 1  (0.0001%) | 0  (0%) | 0  (0%) | 0  (0%) | 3  (0.0003%) | 40  (0.0007%) |
| **Total per year:** | 38  (0.007%) | 9  (0.001%) | 2  (0.0003%) | 1  (0.0001%) | 0  (0%) | 1  (0.00009%) | 6  (0.0006%) | 57  (0.001%) |

**Additional file 1: Table S2:** Incidence rates for the 14 studied postoperative complications stratified into surgical subspecialties.

Values are listed by procedure year as incidence (%) and number of cases

SSI: Surgical Site Infection; UTI: Urinary Tract Infection; DVT: Deep Venous Thrombosis; CVA: Cerebrovascular Accident; OR: Operating Room

| Procedure year | 2012 | 2013 | 2014 | 2015 | 2016 | 2017 | 2018 | All | 2016 | 2017 | 2018 |
| --- | --- | --- | --- | --- | --- | --- | --- | --- | --- | --- | --- |
| **Cardiac surgery:** | | | | | | | | |  |  |  |
| Superficial SSI: | 90 (0.02%) | 68 (0.01%) | 67 (0.009%) | 91 (0.01%) | 98 (0.01%) | 101 (0.01%) | 102 (0.01%) | 617 (0.01%) |  |  |  |
| Deep SSI: | 21 (0.004%) | 13 (0.002%) | 11 (0.001%) | 28 (0.003%) | 19 (0.002%) | 13 (0.001%) | 16 (0.002%) | 121 (0.002%) |  |  |  |
| Organ/Space SSI: | 18 (0.003%) | 11 (0.002%) | 11 (0.001%) | 11 (0.001%) | 21 (0.002%) | 14 (0.001%) | 20 (0.002%) | 106 (0.002%) |  |  |  |
| Pneumonia: | 161 (0.03%) | 163 (0.03%) | 202 (0.03%) | 212 (0.02%) | 257 (0.03%) | 216 (0.02%) | 198 (0.02%) | 1,409 (0.02%) |  |  |  |
| Pulmonary embolism: | 18 (0.003%) | 16 (0.002%) | 19 (0.003%) | 24 (0.003%) | 20 (0.002%) | 28 (0.003%) | 23 (0.002%) | 148 (0.003%) |  |  |  |
| UTI: | 86 (0.02%) | 67 (0.01%) | 78 (0.01%) | 75 (0.008%) | 62 (0.006%) | 56 (0.005%) | 76 (0.007%) | 500 (0.009%) |  |  |  |
| DVT | 60 (0.01%) | 40 (0.006%) | 40 (0.005%) | 48 (0.005%) | 43 (0.004%) | 46 (0.004%) | 53 (0.005%) | 330 (0.006%) |  |  |  |
| CVA/stroke: | 65 (0.01%) | 47 (0.007%) | 59 (0.008%) | 72 (0.008%) | 82 (0.008%) | 81 (0.008%) | 70 (0.007%) | 476 (0.008%) |  |  |  |
| Renal fail: | 74 (0.01%) | 56 (0.009%) | 79 (0.01%) | 70 (0.008%) | 88 (0.009%) | 82 (0.008%) | 68 (0.007%) | 517 (0.009%) |  |  |  |
| Myocardial infarction: | 21 (0.004%) | 20 (0.003%) | 18 (0.002%) | 34 (0.004%) | 34 (0.003%) | 23 (0.002%) | 32 (0.003%) | 182 (0.003%) |  |  |  |
| Cardiac arrest: | 80 (0.01%) | 79 (0.01%) | 87 (0.01%) | 101 (0.01%) | 105 (0.01%) | 119 (0.01%) | 85 (0.008%) | 656 (0.01%) |  |  |  |
| Sepsis: | 75 (0.01%) | 33 (0.005%) | 45 (0.006%) | 50 (0.006%) | 52 (0.005%) | 59 (0.006%) | 44 (0.004%) | 358 (0.006%) |  |  |  |
| Unplanned  return to OR: | 269 (0.05%) | 252 (0.04%) | 249 (0.03%) | 282 (0.03%) | 280 (0.03%) | 245 (0.02%) | 250 (0.02%) | 1,827 (0.03%) |  |  |  |
| Death: | 115 (0.02%) | 89 (0.01%) | 119 (0.02%) | 124 (0.01%) | 124 (0.01%) | 136 (0.01%) | 104 (0.01%) | 881 (0.01%) |  |  |  |
| **General surgery:** | | | | | | | | |  |  |  |
| Superficial SSI: | 6,874 (1.3%) | 7,436 (1.1%) | 8,088 (1.1%) | 8,329 (0.9%) | 8,571 (0.9%) | 8,029 (0.8%) | 7,486 (0.7%) | 54,813 (0.9%) |  |  |  |
| Deep SSI: | 1,950 (0.4%) | 2,430 (0.4%) | 2,809 (0.4%) | 2,861 (0.3%) | 2,148 (0.2%) | 1,938 (0.2%) | 1,911 (0.2%) | 16,047 (0.3%) |  |  |  |
| Organ/Space SSI: | 4,657 (0.9%) | 6,044 (0.9%) | 7,457 (1.0%) | 8,667 (1.0%) | 9,943 (1.0%) | 10,494 (1.0%) | 10,654 (1.0%) | 57,916 (1.0%) |  |  |  |
| Pneumonia: | 3,575 (0.7%) | 4,510 (0.7%) | 5,504 (0.7%) | 6,154 (0.7%) | 6,378 (0.6%) | 5,974 (0.6%) | 5,434 (0.5%) | 37,529 (0.6%) |  |  |  |
| Pulmonary embolism: | 848 (0.2%) | 982 (0.2%) | 1,129 (0.2%) | 1,327 (0.1%) | 1,310 (0.1%) | 1,368 (0.1%) | 1,285 (0.1%) | 8,249 (0.1%) |  |  |  |
| UTI: | 3,783 (0.7%) | 3,803 (0.6%) | 4,199 (0.6%) | 4,451 (0.5%) | 4,370 (0.4%) | 4,179 (0.4%) | 4,031 (0.4%) | 28,816 (0.5%) |  |  |  |
| DVT: | 1,703 (0.3%) | 1,900 (0.3%) | 2,155 (0.3%) | 2,487 (0.3%) | 2,516 (0.3%) | 2,575 (0.3%) | 2,397 (0.2%) | 15,733 (0.3%) |  |  |  |
| CVA/stroke: | 323 (0.06%) | 399 (0.06%) | 469 (0.06%) | 504 (0.06%) | 542 (0.05%) | 558 (0.05%) | 552 (0.05%) | 3,347 (0.06%) |  |  |  |
| Renal fail: | 958 (0.2%) | 1,065 (0.2%) | 1,242 (0.2%) | 1,388 (0.2%) | 1,372 (0.1%) | 1,342 (0.1%) | 1,383 (0.1%) | 8,750 (0.1%) |  |  |  |
| Myocardial infarction: | 795 (0.1%) | 854 (0.1%) | 1,087 (0.1%) | 1,348 (0.2%) | 1,430 (0.1%) | 1,459 (0.1%) | 1,403 (0.1%) | 8,376 (0.1%) |  |  |  |
| Cardiac arrest: | 782 (0.1%) | 981 (0.2%) | 1,187 (0.2%) | 1,390 (0.2%) | 1,472 (0.1%) | 1,485 (0.1%) | 1,400 (0.1%) | 8,697 (0.1%) |  |  |  |
| Sepsis: | 5,479 (1.0%) | 7,468 (1.1%) | 9,685 (1.3%) | 10,574 (1.2%) | 11,636 (1.2%) | 12,062 (1.2%) | 11,409 (1.1%) | 68,313 (1.2%) |  |  |  |
| Unplanned  return to OR: | 8,240 (1.5%) | 9,288 (1.4%) | 10,094 (1.3%) | 11,688 (1.3%) | 11,859 (1.2%) | 11,994 (1.2%) | 11,727 (1.1%) | 74,890(1.3%) |  |  |  |
| Death: | 3,067 (0.6%) | 3,702 (0.6%) | 4,243 (0.6%) | 4,723 (0.5%) | 5,106 (0.5%) | 4,972 (0.5%) | 4,895 (0.5%) | 30,708 (0.5%) |  |  |  |
| **Gynecology:** | | | | | | | | |  |  |  |
| Superficial SSI: | 547 (0.1%) | 606 (0.09%) | 768 (0.1%) | 887 (0.1%) | 1,010 (0.1%) | 1,131 (0.1%) | 1,253 (0.1%) | 6,202 (0.1%) |  |  |  |
| Deep SSI: | 101 (0.02%) | 174 (0.03%) | 208 (0.03%) | 216 (0.02%) | 214 (0.02%) | 177 (0.02%) | 215 (0.02%) | 1,305 (0.02%) |  |  |  |
| Organ/Space SSI: | 301 (0.06%) | 373 (0.06%) | 463 (0.06%) | 613 (0.07%) | 838 (0.08%) | 923 (0.09%) | 1,058 (0.1%) | 4,569 (0.08%) |  |  |  |
| Pneumonia: | 102 (0.02%) | 179 (0.03%) | 199 (0.03%) | 208 (0.02%) | 200 (0.02%) | 215 (0.02%) | 220 (0.02%) | 1,323 (0.02%) |  |  |  |
| Pulmonary embolism: | 110 (0.02%) | 149 (0.02%) | 169 (0.02%) | 192 (0.02%) | 240 (0.02%) | 224 (0.02%) | 235 (0.02%) | 1,319 (0.02%) |  |  |  |
| UTI: | 1,001 (0.2%) | 1,185 (0.2%) | 1,372 (0.2%) | 1,725 (0.2%) | 1,789 (0.2%) | 1,966 (0.2%) | 2,215 (0.2%) | 11,253 (0.2%) |  |  |  |
| DVT: | 93 (0.02%) | 113 (0.02%) | 133 (0.02%) | 114 (0.01%) | 163 (0.02%) | 153 (0.01%) | 162 (0.02%) | 931 (0.02%) |  |  |  |
| CVA/stroke: | 17 (0.003%) | 19 (0.003%) | 19 (0.003%) | 29 (0.003%) | 28 (0.003%) | 29 (0.003%) | 24 (0.002%) | 165 (0.003%) |  |  |  |
| Renal fail: | 11 (0.002%) | 25 (0.004%) | 16 (0.002%) | 29 (0.003%) | 29 (0.003%) | 21 (0.002%) | 36 (0.004%) | 167 (0.003%) |  |  |  |
| Myocardial infarction: | 23 (0.004%) | 23 (0.004%) | 30 (0.004%) | 51 (0.006%) | 81 (0.008%) | 69 (0.007%) | 63 (0.006%) | 340 (0.006%) |  |  |  |
| Cardiac arrest: | 22 (0.003%) | 18 (0.003%) | 25 (0.003%) | 21 (0.002%) | 37 (0.004%) | 47 (0.005%) | 45 (0.004%) | 215 (0.004%) |  |  |  |
| Sepsis: | 234 (0.04%) | 276 (0.04%) | 355 (0.05%) | 369 (0.04%) | 430 (0.04%) | 472 (0.05%) | 506 (0.05%) | 2,642 (0.04%) |  |  |  |
| Unplanned  return to OR: | 578 (0.1%) | 757 (0.1%) | 832 (0.1%) | 998 (0.1%) | 1,189 (0.1%) | 1,290 (0.1%) | 1,307 (0.1%) | 6,951 (0.1%) |  |  |  |
| Death: | 47 (0.009%) | 53 (0.008%) | 67 (0.009%) | 61 (0.007%) | 85 (0.008%) | 73 (0.007%) | 85 (0.008%) | 471 (0.008%) |  |  |  |

| **Neurosurgery:** | | | | | | | | |
| --- | --- | --- | --- | --- | --- | --- | --- | --- |
| Superficial SSI: | 172 (0.03%) | 263 (0.04%) | 297 (0.04%) | 375 (0.04%) | 466 (0.05%) | 477 (0.05%) | 461 (0.05%) | 2,511 (0.04%) |
| Deep SSI: | 110 (0.02%) | 176 (0.03%) | 244 (0.03%) | 273 (0.03%) | 221 (0.02%) | 203 (0.02%) | 207 (0.02%) | 1,434 (0.02%) |
| Organ/Space SSI: | 91 (0.02%) | 125 (0.02%) | 187 (0.02%) | 241 (0.03%) | 305 (0.03%) | 387 (0.04%) | 387 (0.04%) | 1,723 (0.03%) |
| Pneumonia: | 293 (0.05%) | 450 (0.07%) | 520 (0.07%) | 707 (0.08%) | 742 (0.07%) | 791 (0.08%) | 765 (0.07%) | 4,268 (0.07%) |
| Pulmonary embolism: | 139 (0.03%) | 182 (0.03%) | 197 (0.03%) | 261 (0.03%) | 313 (0.03%) | 340 (0.03%) | 294 (0.03%) | 1,726 (0.03%) |
| UTI: | 430 (0.08%) | 514 (0.08%) | 581 (0.08%) | 654 (0.07%) | 772 (0.08%) | 806 (0.08%) | 764 (0.07%) | 4,521 (0.08%) |
| DVT: | 241 (0.04%) | 327 (0.05%) | 365 (0.05%) | 469 (0.05%) | 525 (0.05%) | 557 (0.05%) | 497 (0.05%) | 2,981 (0.05%) |
| CVA/stroke: | 120 (0.02%) | 159 (0.02%) | 169 (0.02%) | 215 (0.02%) | 293 (0.03%) | 279 (0.03%) | 297 (0.03%) | 1,532 (0.03%) |
| Renal fail: | 27 (0.005%) | 35 (0.005%) | 61 (0.008%) | 55 (0.006%) | 58 (0.006%) | 63 (0.006%) | 69 (0.007%) | 368 (0.006%) |
| Myocardial infarction: | 59 (0.01%) | 58 (0.009%) | 94 (0.01%) | 135 (0.02%) | 153 (0.02%) | 164 (0.02%) | 134 (0.01%) | 797 (0.01%) |
| Cardiac arrest: | 50 (0.009%) | 81 (0.01%) | 92 (0.01%) | 114 (0.01%) | 129 (0.01%) | 139 (0.01%) | 139 (0.01%) | 744 (0.01%) |
| Sepsis: | 219 (0.04%) | 294 (0.05%) | 400 (0.05%) | 456 (0.05%) | 504 (0.05%) | 552 (0.05%) | 553 (0.05%) | 2,978 (0.05%) |
| Unplanned  return to OR: | 863 (0.2%) | 1,153 (0.2%) | 1,437 (0.2%) | 1,770 (0.2%) | 1,844 (0.2%) | 1,928 (0.2%) | 1,918 (0.2%) | 10,913 (0.2%) |
| Death: | 297 (0.05%) | 365 (0.06%) | 416 (0.06%) | 537 (0.06%) | 636 (0.06%) | 643 (0.06%) | 645 (0.06%) | 3,539 (0.06 %) |
| **Orthopedic surgery:** | | | | | | | | |
| Superficial SSI: | 644 (0.1%) | 762 (0.1%) | 922 (0.1%) | 1,107 (0.1%) | 1,301 (0.1%) | 1,356 (0.1%) | 1,419 (0.1%) | 7,511 (0.1%) |
| Deep SSI: | 331 (0.06%) | 479 (0.07%) | 650 (0.09%) | 720 (0.08%) | 701 (0.07%) | 672 (0.07%) | 636 (0.06%) | 4,189 (0.07%) |
| Organ/Space SSI: | 242 (0.04%) | 348 (0.05%) | 489 (0.07%) | 664 (0.07%) | 1,081 (0.1%) | 1,170 (0.1%) | 1,261 (0.1%) | 5,255 (0.09%) |
| Pneumonia: | 598 (0.1%) | 902 (0.1%) | 1,209 (0.2%) | 1,637 (0.2%) | 1,743 (0.2%) | 1,694 (0.2%) | 1,686 (0.2%) | 9,469 (0.2%) |
| Pulmonary embolism: | 373 (0.07%) | 476 (0.07%) | 613 (0.08%) | 808 (0.09%) | 801 (0.08%) | 848 (0.08%) | 854 (0.08%) | 4,773 (0.08%) |
| UTI: | 1,358 (0.2%) | 1,641 (0.3%) | 1,655 (0.2%) | 2,201 (0.2%) | 2,329 (0.2%) | 2,281 (0.2%) | 2,410 (0.2%) | 13,875 (0.2%) |
| DVT: | 542 (0.1%) | 754 (0.1%) | 914 (0.1%) | 1,140 (0.1%) | 1,300 (0.1%) | 1,310 (0.1%) | 1,246 (0.1%) | 7,206 (0.1%) |
| CVA/stroke: | 111 (0.02%) | 169 (0.03%) | 193 (0.03%) | 283 (0.03%) | 353 (0.04%) | 358 (0.04%) | 325 (0.03%) | 1,792 (0.03%) |
| Renal fail: | 102 (0.02%) | 116 (0.02% | 147 (0.02%) | 180 (0.02%) | 238 (0.02%) | 221 (0.02%) | 250 (0.02%) | 1,254 (0.02%) |
| Myocardial infarction: | 319 (0.06%) | 349 (0.05%) | 444 (0.06%) | 754 (0.09%) | 863 (0.09%) | 843 (0.08%) | 820 (0.08%) | 4,392 (0.07%) |
| Cardiac arrest: | 144 (0.03%) | 170 (0.03%) | 253 (0.03%) | 320 (0.04%) | 358 (0.04%) | 387 (0.04%) | 383 (0.04%) | 2,015 (0.03%) |
| Sepsis: | 454 (0.1%) | 577 (0.09%) | 934 (0.1%) | 1,128 (0.1%) | 1,357 (0.1%) | 1,440 (0.1%) | 1,436 (0.1%) | 7,326 (0.1%) |
| Unplanned  return to OR: | 1,670 (0.3%) | 2,097 (0.3%) | 2,744 (0.4%) | 3,661 (0.4%) | 4,260 (0.4%) | 4,058 (0.4%) | 4,346 (0.4%) | 22,836 (0.4%) |
| Death: | 667 (0.1%) | 816 (0.1%) | 1,112 (0.1%) | 1,566 (0.2%) | 1,696 (0.2%) | 1,723 (0.2%) | 1,882 (0.2%) | 9,462 (0.2%) |
| **Otolaryngology (ENT):** | | | | | | | | |
| Superficial SSI: | 140 (0.03%) | 179 (0.03%) | 200 (0.03%) | 240 (0.03%) | 253 (0.03%) | 254 (0.02%) | 275 (0.03%) | 1,541 (0.03%) |
| Deep SSI: | 56 (0.01%) | 83 (0.01%) | 80 (0.01%) | 131 (0.01%) | 125 (0.01%) | 103 (0.01%) | 87 (0.009%) | 665 (0.01%) |
| Organ/Space SSI: | 43 (0.008%) | 56 (0.009%) | 84 (0.01%) | 95 (0.01%) | 122 (0.01%) | 138 (0.01%) | 121 (0.01%) | 659 (0.01%) |
| Pneumonia: | 76 (0.01%) | 102 (0.02%) | 146 (0.02%) | 175 (0.02%) | 151 (0.02%) | 173 (0.02%) | 161 (0.02%) | 984 (0.02%) |
| Pulmonary embolism: | 16 (0.003%) | 22 (0.003%) | 26 (0.003%) | 21 (0.002%) | 33 (0.003%) | 31 (0.003%) | 33 (0.003%) | 182 (0.003%) |
| UTI: | 53 (0.01%) | 46 (0.007%) | 91 (0.01%) | 82 (0.009%) | 101 (0.01%) | 81 (0.008%) | 99 (0.01%) | 553 (0.009%) |
| DVT: | 25 (0.005%) | 33 (0.005%) | 29 (0.004%) | 40 (0.005%) | 48 (0.005%) | 45 (0.004%) | 51 (0.005%) | 271 (0.005%) |
| CVA/stroke: | 14 (0.002%) | 10 (0.002%) | 18 (0.002%) | 26 (0.003%) | 22 (0.002%) | 24 (0.002%) | 18 (0.002%) | 132 (0.002%) |
| Renal fail: | 1 (0.0002%) | 6 (0.0009%) | 6 (0.0008%) | 7 (0.0008%) | 6 (0.0006%) | 13 (0.001%) | 8 (0.0008%) | 47 (0.0008%) |
| Myocardial infarction: | 16 (0.003%) | 26 (0.004%) | 27 (0.004%) | 28 (0.003%) | 31 (0.003%) | 41 (0.004%) | 34 (0.003%) | 203 (0.003%) |
| Cardiac arrest: | 11 (0.002%) | 19 (0.003%) | 20 (0.003%) | 26 (0.003%) | 20 (0.002%) | 20 (0.002%) | 24 (0.002%) | 140 (0.002%) |
| Sepsis: | 61 (0.01%) | 84 (0.01%) | 128 (0.02%) | 116 (0.01%) | 133 (0.01%) | 149 (0.01%) | 123 (0.01%) | 794 (0.01%) |
| Unplanned  return to OR: | 413 (0.08%) | 506 (0.08%) | 622 (0.08%) | 732 (0.08%) | 815 (0.08%) | 771 (0.07%) | 773 (0.08%) | 4,632 (0.08%) |
| Death: | 31 (0.006%) | 31 (0.005%) | 31 (0.004%) | 58 (0.007%) | 46 (0.005%) | 56 (0.005%) | 38 (0.004%) | 291 (0.005%) |

| **Plastics:** | | | | | | | | |
| --- | --- | --- | --- | --- | --- | --- | --- | --- |
| Superficial SSI: | 316 (0.06%) | 340 (0.05%) | 348 (0.05%) | 429 (0.05%) | 558 (0.06%) | 595 (0.06%) | 635 (0.06%) | 3,221 (0.06%) |
| Deep SSI: | 123 (0.02%) | 159 (0.02%) | 172 (0.02%) | 175 (0.02%) | 200 (0.02%) | 232 (0.02%) | 208 (0.02%) | 1,269 (0.02%) |
| Organ/Space SSI: | 86 (0.02%) | 71 (0.01%) | 74 (0.01%) | 138 (0.02%) | 212 (0.02%) | 244 (0.02%) | 264 (0.03%) | 1,089 (0.02%) |
| Pneumonia: | 36 (0.007%) | 38 (0.006%) | 45 (0.006%) | 73 (0.008%) | 75 (0.008%) | 75 (0.007%) | 87 (0.009%) | 429 (0.007%) |
| Pulmonary embolism: | 23 (0.004%) | 26 (0.004%) | 37 (0.005%) | 44 (0.005%) | 41 (0.004%) | 47 (0.005%) | 49 (0.005%) | 267 (0.005%) |
| UTI: | 86 (0.02%) | 57 (0.009%) | 68 (0.009%) | 104 (0.01%) | 134 (0.01%) | 114 (0.01%) | 130 (0.01%) | 693 (0.01%) |
| DVT: | 39 (0.007%) | 50 (0.008%) | 43 (0.006%) | 54 (0.006%) | 56 (0.006%) | 69 (0.007%) | 74 (0.007%) | 385 (0.007%) |
| CVA/stroke: | 6 (0.001%) | 7 (0.001%) | 6 (0.0008%) | 11 (0.001%) | 11 (0.001%) | 10 (0.001%) | 2 (0.0002%) | 53 (0.0009%) |
| Renal fail: | 9 (0.002%) | 10 (0.002%) | 14 (0.002%) | 19 (0.002%) | 12 (0.001%) | 12 (0.001%) | 8 (0.0008%) | 84 (0.001%) |
| Myocardial infarction: | 9 (0.002%) | 7 (0.001%) | 9 (0.001%) | 17 (0.002%) | 17 (0.002%) | 23 (0.002%) | 19 (0.002%) | 101 (0.002%) |
| Cardiac arrest: | 1 (0.0002%) | 6 (0.0009%) | 14 (0.002%) | 15 (0.002%) | 15 (0.001%) | 18 (0.002%) | 18 (0.002%) | 87 (0.001%) |
| Sepsis: | 82 (0.02%) | 101 (0.02%) | 107 (0.01%) | 137 (0.02%) | 169 (0.02%) | 223 (0.02%) | 193 (0.02%) | 1,012 (0.02%) |
| Unplanned  return to OR: | 579 (0.1%) | 734 (0.1%) | 744 (0.1%) | 820 (0.09%) | 1,027 (0.1%) | 1,162 (0.1%) | 1,184 (0.1%) | 6,250 (0.1%) |
| Death: | 15 (0.003%) | 20 (0.003%) | 31 (0.004%) | 37 (0.004%) | 40 (0.004%) | 35 (0.003%) | 39 (0.004%) | 217 (0.004%) |
| **Thoracic:** | | | | | | | | |
| Superficial SSI: | 90 (0.02%) | 105 (0.02%) | 126 (0.02%) | 95 (0.01%) | 143 (0.01%) | 121 (0.01%) | 155 (0.02%) | 835 (0.01%) |
| Deep SSI: | 25 (0.005%) | 36 (0.006%) | 43 (0.006%) | 37 (0.004%) | 22 (0.002%) | 28 (0.003%) | 17 (0.002%) | 208 (0.004%) |
| Organ/Space SSI: | 79 (0.01%) | 130 (0.02%) | 118 (0.02%) | 135 (0.02%) | 215 (0.02%) | 241 (0.02%) | 208 (0.02%) | 1,126 (0.02%) |
| Pneumonia: | 430 (0.08%) | 568 (0.09%) | 631 (0.08%) | 665 (0.08%) | 676 (0.07%) | 760 (0.07%) | 651 (0.06%) | 4,381 (0.07%) |
| Pulmonary embolism: | 51 (0.009%) | 60 (0.009%) | 52 (0.007%) | 71 (0.008%) | 99 (0.01%) | 81 (0.008%) | 69 (0.007%) | 483 (0.008%) |
| UTI: | 106 (0.02%) | 113 (0.02%) | 122 (0.02%) | 114 (0.01%) | 135 (0.01%) | 114 (0.01%) | 109 (0.01%) | 813 (0.01%) |
| DVT: | 85 (0.02%) | 93 (0.01%) | 107 (0.01%) | 95 (0.01%) | 133 (0.01%) | 119 (0.01%) | 96 (0.009%) | 728 (0.01 %) |
| CVA/stroke: | 22 (0.004%) | 27 (0.004%) | 37 (0.005%) | 29 (0.003%) | 44 (0.004%) | 54 (0.005%) | 45 (0.004%) | 258 (0.004%) |
| Renal fail: | 40 (0.007%) | 52 (0.008%) | 43 (0.006%) | 62 (0.007%) | 63 (0.006%) | 57 (0.006%) | 65 (0.006%) | 382 (0.006%) |
| Myocardial infarction: | 22 (0.004%) | 43 (0.007%) | 47 (0.006%) | 60 (0.007%) | 62 (0.006%) | 65 (0.006%) | 67 (0.007%) | 366 (0.006%) |
| Cardiac arrest: | 61 (0.01%) | 78 (0.01%) | 78 (0.01%) | 79 (0.009%) | 87 (0.009%) | 98 (0.01%) | 106 (0.01%) | 587 (0.01%) |
| Sepsis: | 164 (0.03%) | 182 (0.03%) | 252 (0.03%) | 228 (0.03%) | 281 (0.03%) | 288 (0.03%) | 247 (0.02%) | 1,642 (0.03%) |
| Unplanned  return to OR: | 360 (0.06%) | 496 (0.08%) | 427 (0.06%) | 486 (0.05%) | 597 (0.06%) | 590 (0.06%) | 618 (0.06%) | 3,574 (0.06%) |
| Death: | 187 (0.03%) | 198 (0.03%) | 221 (0.03%) | 214 (0.02%) | 243 (0.02%) | 240 (0.02%) | 248 (0.02%) | 1551 (0.03%) |
| **Urology:** | | | | | | | | |
| Superficial SSI: | 246 (0.05%) | 294 (0.05%) | 350 (0.05%) | 383 (0.04%) | 486 (0.05%) | 500 (0.05%) | 469 (0.05%) | 2,728 (0.05%) |
| Deep SSI: | 58 (0.01%) | 89 (0.01%) | 99 (0.01%) | 103 (0.01%) | 109 (0.01%) | 78 (0.008%) | 80 (0.008%) | 616 (0.01%) |
| Organ/Space SSI: | 141 (0.03%) | 198 (0.03%) | 258 (0.03%) | 370 (0.04%) | 499 (0.05%) | 629 (0.06%) | 573 (0.06%) | 2,668 (0.05%) |
| Pneumonia: | 190 (0.03%) | 228 (0.03%) | 286 (0.04%) | 346 (0.04%) | 416 (0.04%) | 425 (0.04%) | 364 (0.04%) | 2,255 (0.04%) |
| Pulmonary embolism: | 116 (0.02%) | 126 (0.02%) | 159 (0.02%) | 195 (0.02%) | 237 (0.02%) | 245 (0.02%) | 236 (0.02%) | 1,314 (0.02%) |
| UTI: | 897 (0.2%) | 1,108 (0.2%) | 1,206 (0.2%) | 1,538 (0.2%) | 1,824 (0.1%) | 2,029 (0.2%) | 2,040 (0.2%) | 10,642 (0.2%) |
| DVT: | 164 (0.03%) | 201 (0.03%) | 203 (0.03%) | 272 (0.03%) | 326 (0.03%) | 335 (0.03%) | 317 (0.03%) | 1,818 (0.03%) |
| CVA/stroke: | 33 (0.006%) | 42 (0.006%) | 52 (0.007%) | 74 (0.008%) | 71 (0.007%) | 77 (0.007%) | 76 (0.007%) | 425 (0.007%) |
| Renal fail: | 80 (0.02%) | 106 (0.02%) | 125 (0.02%) | 145 (0.02%) | 192 (0.02%) | 182 (0.02%) | 157 (0.02%) | 987 (0.02%) |
| Myocardial infarction: | 104 (0.02%) | 107 (0.02%) | 147 (0.02%) | 157 (0.02%) | 205 (0.02%) | 245 (0.02%) | 187 (0.02%) | 1,152 (0.02%) |
| Cardiac arrest: | 50 (0.009%) | 68 (0.01%) | 69 (0.009%) | 109 (0.01%) | 132 (0.01%) | 131 (0.01%) | 113 (0.01%) | 672 (0.01%) |
| Sepsis: | 320 (0.06%) | 414 (0.06%) | 508 (0.07%) | 567 (0.06%) | 696 (0.07%) | 848 (0.08%) | 810 (0.08%) | 4,163 (0.07%) |
| Unplanned  return to OR: | 586 (0.1%) | 655 (0.1%) | 809 (0.1%) | 1,075 (0.1%) | 1,243 (0.1%) | 1,347 (0.1%) | 1,243 (0.1%) | 6,958 (0.1%) |
| Death: | 163 (0.03%) | 177 (0.03%) | 188 (0.03%) | 244 (0.03%) | 302 (0.03%) | 290 (0.03%) | 275 (0.03%) | 1,639 (0.03%) |

| **Vascular:** | | | | | | | | |
| --- | --- | --- | --- | --- | --- | --- | --- | --- |
| Superficial SSI: | 943 (0.2%) | 1,012 (0.2%) | 1,029 (0.1%) | 1,136 (0.1%) | 1,191 (0.1%) | 1,149 (0.1%) | 1,014 (0.1%) | 7,474 (0.1%) |
| Deep SSI: | 374 (0.07%) | 482 (0.07%) | 530 (0.07%) | 541 (0.06%) | 467 (0.05%) | 516 (0.05%) | 402 (0.04%) | 3,312 (0.06%) |
| Organ/Space SSI: | 117 (0.02%) | 186 (0.03%) | 164 (0.02%) | 212 (0.02%) | 342 (0.03%) | 424 (0.04%) | 440 (0.04%) | 1,885 (0.03%) |
| Pneumonia: | 734 (0.1%) | 912 (0.1%) | 990 (0.1%) | 1,133 (0.1%) | 1,193 (0.1%) | 1,126 (0.1%) | 932 (0.09%) | 7,020 (0.1%) |
| Pulmonary embolism: | 106 (0.02%) | 93 (0.01%) | 113 (0.02%) | 123 (0.01%) | 135 (0.01%) | 155 (0.02%) | 101 (0.01%) | 826 (0.01%) |
| UTI: | 666 (0.1%) | 628 (0.1%) | 624 (0.08%) | 662 (0.07%) | 650 (0.06%) | 601 (0.06%) | 471 (0.05%) | 4,302 (0.07%) |
| DVT | 363 (0.07%) | 376 (0.06%) | 347 (0.05%) | 371 (0.04%) | 401 (0.04%) | 417 (0.04%) | 349 (0.03%) | 2,624 (0.05%) |
| CVA/stroke: | 372 (0.07%) | 439 (0.07%) | 392 (0.05%) | 499 (0.06%) | 535 (0.05%) | 500 (0.05%) | 434 (0.04%) | 3,171 (0.05%) |
| Renal fail: | 445 (0.06%) | 475 (0.07%) | 445 (0.06%) | 455 (0.05%) | 519 (0.05%) | 476 (0.05%) | 404 (0.04%) | 3,219 (0.05%) |
| Myocardial infarction: | 629 (0.1%) | 614 (0.09%) | 714 (0.1%) | 754 (0.09%) | 916 (0.09%) | 921 (0.09%) | 848 (0.08%) | 5,396 (0.09%) |
| Cardiac arrest: | 415 (0.08%) | 503 (0.08%) | 526 (0.07%) | 649 (0.07% | 602 (0.06%) | 612 (0.06%) | 546 (0.05%) | 3,853 (0.07%) |
| Sepsis: | 662 (0.1%) | 740 (0.1%) | 891 (0.1%) | 968 (0.1%) | 1,108 (0.1%) | 1,157 (0.1%) | 975 (0.1%) | 6,501 (0.1%) |
| Unplanned  return to OR: | 3,425 (0.6%) | 3,792 (0.6%) | 3,864 (0.5%) | 4,363 (0.5%) | 4,434 (0.4%) | 4,531 (0.4%) | 3,894 (0.4%) | 28,303 (0.5%) |
| Death: | 1,051 (0.2%) | 1,305 (0.2%) | 1,321 (0.2%) | 1,419 (0.2%) | 1,454 (0.1%) | 1,496 (0.1%) | 1,335 (0.1%) | 9,381 (0.2%) |
